# Simultaneous monteggia type I fracture equivalent with ipsilateral fracture of the distal radius and ulna in a child: a case report

**DOI:** 10.1186/1752-1947-2-190

**Published:** 2008-06-02

**Authors:** Asheesh Sood, Osman Khan, Tajesh Bagga

**Affiliations:** 1 Department of Orthopaedics and Trauma, Diana Princess of Wales Hospital, Grimsby, UK

## Abstract

**Introduction:**

Simultaneous Monteggia injuries of the elbow and ipsilateral distal radius and ulna fractures are very rare.

**Case Presentation:**

A unique case of a type I Monteggia fracture equivalent with ipsilateral fracture of the distal radius and ulna (Salter-Harris type II) in a child is reported. We describe the management of this unique fracture and discuss the possible mechanism of injury.

**Conclusion:**

We have highlighted a rare combination of injuries. Early recognition and prompt surgical intervention can lead to a satisfactory outcome even in these complex injuries.

## Introduction

The term 'Monteggia lesion' is applied to all forearm injuries that have a dislocation of the radial head and fracture of the ulna. This injury is relatively uncommon in children. We report a unique case of a type I Monteggia fracture equivalent [1] with ipsilateral fracture of the distal radius and ulna in a child. To the best of the authors' knowledge, there have been no reports in the literature of cases with exactly the same combination of injuries.

## Case presentation

An 11-year-old girl fell off a swing 1.8 m high and injured her left forearm. On examination she had a severe dorsal angular deformity of the wrist and the ipsilateral elbow was also very swollen. There was no neurovascular deficit.

**Figure 1:**
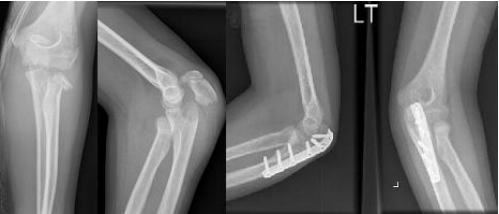
Radiographs showing a Monteggia type I equivalent elbow injury before and after internal fixation.

**Figure 2:**
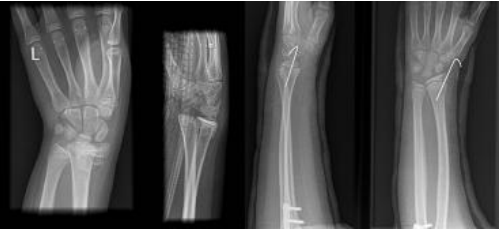
Radiographs showing a Salter-Harris type II distal radius fracture before and after wiring.

Radiographs of the elbow and wrist revealed a complete fracture of the olecranon with a fracture of the radial neck (Figure 1), and a Salter-Harris type II fracture of the distal radius and ulna with complete displacement (Figure 2). The arm was immobilised in an above-elbow slab and the patient was taken to the operating theatre the following day.

Under general anaesthesia using a dorsolateral approach, the ulna was reduced and fixed using a six-hole dynamic compression plate (DCP); see Figure 1. The radial neck was reduced under direct vision. The wrist fracture was manipulated under image intensifier control and a satisfactory reduction was achieved. The arm was immobilised in an above-elbow slab in 90° of flexion in a mid-prone position.

Postoperatively she developed symptoms of median nerve compression, complaining of tingling and numbness in the index and middle finger. After a 12-hour period of observation, with the limb elevated in a Bradford sling, the symptoms did not resolve. We considered the options: a further period of observation or surgical intervention (performing a carpal tunnel decompression). The latter was favoured on clinical grounds, electrophysiological studies were not performed. She underwent urgent carpal tunnel decompression followed by manipulation and Kirschner (K)-wire stabilisation of the wrist. The median nerve symptoms resolved after surgery. She made a satisfactory postoperative recovery and was discharged the following day.

**Figure 3:**
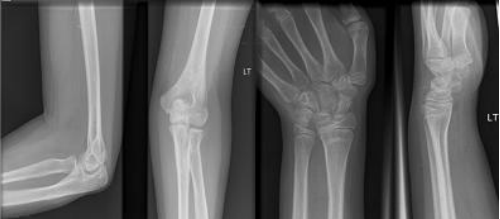
Radiographs of the elbow and wrist after removal of the metalwork.

At a follow-up 6 weeks later, the fracture had united both clinically and radiologically. She had a good range of motion in all fingers and sensation had returned to the index and middle finger. The plaster and wire were removed (Figure 3) and physiotherapy commenced. At 3 months, the metalwork from the elbow was removed (Figure 3). The range of motion at the elbow was 30° to 110°. At 4 months, the range of motion had further improved to 5° to 120° and had achieved full pronation and supination. At 7 months, she had recovered complete range of motion in both elbow and wrist.

## Discussion

While fractures of the distal forearm are quite common in children, the Monteggia lesion remains uncommon. Simultaneous ipsilateral proximal and distal forearm fractures are very rare. Previous such combinations reported include: type III Monteggia injury with ipsilateral distal radius and ulna fractures [2]; olecranon fracture and distal radial epiphysis [3]; type II Monteggia fracture with fracture separation of the distal radial physis [4]; type IV Monteggia injury with distal diaphyseal fracture of the radius [5]; 11 cases of Monteggia fracture dislocation with fracture of the ipsilateral radius and ulna [6]; three epiphyseal fractures (distal radius and ulna and proximal radius) and a diaphyseal ulnar fracture in the same forearm [7].

Four types of Monteggia fractures as well as three equivalent types have been described [1]. These were described according to the direction of the radial head subluxation: the most common (75%) is fracture of the proximal third ulna, anterior angulation of the fracture with anterior dislocation of the radial head; the second most common is fracture of the proximal third of the ulna, lateral angulation of the fracture and lateral dislocation of the radial head; proximal ulna fracture with post-dislocation of the radial head; fracture of the proximal radius and ulna with dislocation of the radial head.

Three Monteggia equivalent fractures have been described: isolated radial head dislocation; fractures of the proximal ulna with fracture of the radial neck (our case report); proximal one-third fracture of both bones with radial fracture proximal to an ulna fracture.

Closed reduction is generally the treatment of choice for Monteggia fractures in children [3,4]. Quite often in Monteggia equivalent fractures proper alignment cannot be obtained and open reduction may be necessary. In this particular instance, we felt that we would be unable to achieve satisfactory closed reduction and proceeded to open reduction. The radial neck was reduced under direct vision and the ulna was internally fixed using a narrow DCP.

In the Monteggia type II equivalent reported by Osada et al. [7], the child sustained three epiphyseal fractures (distal radius and ulna and proximal radius) and a diaphyseal (mid-shaft) ulnar fracture. The authors explained the difficulty they had in attempting a closed reduction, and subsequently opted for a minimally invasive internal fixation. During the open reduction, three of the four fractures were secured with K-wires. The midshaft ulna fracture was stabilised with K-wires reinforced with a circular soft wire. This led to the ulna shaft remaining posteriorly convex, in turn leading to posterior convexity of the radial neck. The authors recommended that in unstable forearm fractures in children, diaphyseal fractures of the ulna should be plated, as in adults.

The mechanism of injury causing simultaneous two level fractures in the forearm is not well understood. When a child has a fall on the outstretched hand, the forearm is in pronation [8]. This original injury leads to fracture separation of the radial physis [4]. The trunk continues to rotate and this combined with longitudinal compression of the wrist leads to the Monteggia lesion.

The child had developed median nerve symptoms after the initial manipulation and the decision to proceed with open carpal tunnel decompression was made purely on clinical grounds. The alternative was to continue observation. However, the latter may have been too distressing for both mother and child. The literature reports that carpal tunnel pressure increases after distal radius fractures, secondary to oedema and bleeding [9]. It rises further if local anaesthetic is introduced into the fracture haematoma, and even higher pressures are recorded with volar flexion of the wrist.

This report highlights an extremely rare injury occurring in combination with a Salter-Harris type II epiphyseal separation at the lower end of the radius. The awareness of this possible injury combination is important to avoid missing a second lesion, which may be hidden by the more significant injury. The lower end of the forearm must be included in the initial radiographic examination. Early recognition and prompt surgical intervention can lead to a good result despite the rarity and seriousness of this injury.

## Conclusion

Our case report has highlighted a rare combination of injuries. While it is true that such injuries occur rarely, one must always be aware of the possibility of associated wrist injuries while dealing with elbow trauma. Thorough clinical and radiological examination is the key to avoid missing such injuries.

## Abbreviations

DCP: dynamic compression plate; K: Kirschner.

## Competing interests

The authors declare that they have no competing interests.

## Authors' contributions

AS carried out the literature search and wrote the manuscript, OK helped in the literature search, collected the X-rays and obtained the patient's consent, TB contributed to the discussion section and edited the manuscript.

## Consent

Written informed consent was obtained from the patient's next-of-kin for publication of this case report and any accompanying images. A copy of the written consent is available for review by the Editor-in-Chief of this journal
